# Avocado (*Persea americana* Mill.) Genomic Divergence Across the Victoria, Tanganyika and Rukwa Lake Basins of Tanzania

**DOI:** 10.3390/ijms27219699

**Published:** 2026-10-29

**Authors:** Andrés J. Cortés, Juma M. Hussein, Ibrahim Juma

**Affiliations:** 1 Department of Plant Breeding, Swedish University of Agricultural Sciences, 23436 Lomma, Sweden; 2 Department of Molecular Biology and Biotechnology, University of Dar es Salaam, Dar es Salaam P.O. Box 35179, Tanzania; 3 Department of Food Science and Technology, University of Dar es Salaam, Dar es Salaam P.O. Box 35134, Tanzania; vuga.ibrahim@udsm.ac.tz

**Keywords:** East Africa, Lake Zone, Western Rift, *in situ* conservation, ‘plus trees’, lcWGS

## Abstract

Avocado (*Persea americana* Mill.) is a crucial horticultural tree crop for food security and rural income in Tanzania. Despite its importance, the genomic divergence of avocado populations in the country’s Lake Zone, Western Zone, and the Southern Highlands region of Rukwa has not previously been investigated. While the Southern Highlands are recognized as a major production hub, a significant knowledge gap exists regarding the genomic diversity, population stratification and racial ancestry of landraces in the Western Rift and Lake Victoria Basin, areas characterized by unique microclimates and historical transboundary trade routes with neighboring Great Lakes nations. Therefore, this study aimed to characterize the genomic diversity of avocado germplasm through low-coverage whole-genome resequencing (lcWGS) of 105 trees from five key regions: Mara, Mwanza and Kagera in the Lake Zone and Lake Victoria Basin; Kigoma in the Western Zone and Lake Tanganyika Basin of the Western Rift; and Rukwa in the Southern Highlands’ Lake Rukwa Basin adjacent to the Lake Tanganyika Basin and Western Rift. The study sought to identify patterns of local differentiation and gene flow. We hypothesized that the avocado populations in these regions are not panmictic but are structured by geographic and ecological barriers, where *(i)* regions bordering the Albertine Rift (i.e., Kigoma and Kagera) exhibit distinct West Indian types likely shaped by transboundary exchange and climate heterogeneity and *(ii)* the Lake Victoria Eastern Basin regions of Mwanza and Mara harbor unique intermediate genotypes resulting from inter-racial hybridization with lowland genotypes. The lcWGS screening generated more than 63,577,355 SNPs across a total of 405 studied trees, after compilation with published lcWGS reads from 300 avocado trees from Tanzanian Morogoro and the Southern Highlands of Iringa, Mbeya and Ruvuma (95) and racial control groups in the Americas (205). Phylogenetic, unsupervised principal component and ADMIXTURE analyses found that germplasm from Rukwa clustered with Southern Highlands germplasm from Iringa, Mbeya, and Ruvuma. In contrast, accessions from Kigoma and Kagera formed a distinct Western Rift lineage enriched for West Indian ancestry, and populations in Mara and Mwanza occupied intermediate positions, suggesting extensive inter-racial admixture in the eastern Lake Victoria Basin. These genetic clusters may guide the utilization of East African avocado landraces.

## 1. Introduction

The vast eco-geographical richness of East Africa is a source of untapped biological variation [1,2,3] and local agrobiodiversity [4]. Specifically, the regions of Mara, Mwanza, and Kagera (the Lake Zone regions) in northern and northwestern Tanzania, together with Kigoma (the Western Zone region) and Rukwa (the Southern Highlands region) in the country’s western and southwestern parts, respectively, form a unique agroecological corridor shaped by the intersection of the Lake Tanganyika, Lake Victoria and Lake Rukwa basins and the western branch of the Great Rift Valley (i.e., Western or Albertine Rift) [5]. This mosaic of island-like microclimates may drive genetic diversity in perennial fruit trees like avocado (*Persea americana* Mill.), a commodity crop in East Africa [6].

Particularly, the presence of Lake Victoria to the north and Lake Tanganyika to the west could exert a lacustrine effect on the surrounding terrestrial ecosystems [7]. These massive water bodies stabilize temperatures and maintain high relative humidity levels throughout the year. In regions like Kagera and Mwanza, on the western and southern shores of Lake Victoria, this constant moisture mimics the humid subtropical conditions found in avocado’s center of origin in Mesoamerica (spanning the Mexican, Guatemalan and West Indian races) [8,9,10,11,12,13,14] and northwest South America [15,16,17,18] (recently described as the Colombian group [19]), enabling the naturalization and survival of diverse avocado germplasm introduced through continental and maritime trade routes [20,21].

On the other hand, the dramatic topography of the Western Rift Valley, particularly in Kigoma and Rukwa, provides steep altitudinal gradients for local adaptation. Variation in elevation (ranging from ca. 700 m at lake shores to over 2000 m in the highlands) creates distinct vertical life zones over short geographic distances [22,23,24]. Such environmental heterogeneity may in turn impose divergent selection pressures on avocado populations, potentially filtering for a mixture enriched in provenances from the West Indian (lowland-adapted) and the northwest South American (Andean and Caribbean) groups [25].

Similarly, regarding the edaphic and hydrological diversity in the area, these regions transition geologically from the ancient Precambrian rocks of the Tanzania Craton to younger volcanic and lacustrine sediments [26]. The fertile, well-drained volcanic soils of the Rift escarpments, combined with the alluvial deposits of the lake basins, provide an ideal edaphic substrate for avocado root development [27]. This soil mosaic, coupled with bimodal rainfall patterns (with major rains in April and November), has likely fostered a wide range of phenological adaptations and avocado quality traits among local landraces. In short, the western and Lake Zone regions of Tanzania offer an evolutionary laboratory to study racial divergence [28,29,30,31] and resilience to shifting abiotic [32,33,34] and biotic [35,36,37,38,39,40,41,42] pressures. Despite this and the growing nutritional [43,44,45,46,47] and commercial [6,48] value of avocado trading in East Africa, the genomic resources of Tanzania’s avocado western germplasm remain largely uncharacterized. Diversity studies have focused on orchards in the Southern [49,50,51] and Northern [52,53,54,55] Highlands, leaving a research gap regarding the genetic identity of landraces in the Victoria, Tanganyika and Rukwa Lake basins and the Western Rift Valley, where a unique agroecological creole results from lacustrine microclimates, rift-borne soil types and ancient trade routes with neighboring countries. Therefore, the goal of this study was to evaluate the genomic diversity and population stratification of avocado germplasm in these regions using low-coverage whole-genome resequencing (lcWGS). We hypothesized that: *(i)* avocado populations would show genetic stratification likely driven by the sharp geographical gradients of the Rift system, *(ii)* the northwestern region of Kagera and the western region of Kigoma would have a higher frequency of West Indian alleles due to possible inflow and ecological filtering from the Albertine Rift, and *(iii*) the lacustrine environments of Mwanza and Mara could harbor intercrossed clades distinct from the southern hubs.

## 2. Results

Genome-wide SNP marker data called from the lcWGS reads gathered across the 105 newly screened Tanzanian accessions, together with 95 previously assessed trees from the Tanzania Southern Highlands and Morogoro [51] and 205 racial and commercial controls from the Americas [17], were analyzed using complementary phylogenetic, unsupervised multivariate, and ADMIXTURE-based population structure inference approaches. Tanzanian avocado germplasm exhibited geographically structured genetic differentiation rather than panmixia. Specifically, Rukwa accessions consistently clustered with the Southern Highlands, indicating a shared highland genetic ancestry, whereas germplasm from Kigoma and Kagera exhibited closer affinities with the West Indian racial group. In contrast, accessions from Mara and Mwanza occupied more intermediate positions, characterized by putative inter-racial admixture, likely supporting local diversification through race-level hybridization. These results revealed that avocado genetic diversity across western Tanzania and the Lake Zone is shaped by regional structure, contrasting levels of racial ancestry and localized patterns of admixture.

### 2.1. lcWGS-Based SNP Markers

The lcWGS of the 105 sampled Tanzanian avocado trees (Table S1) from the regions of Rukwa, Kigoma, Kagera, Mara and Mwanza yielde on average 29,973,673 reads and 4,526,024,596 bp per sample, with a mean G:C content of 40.14%, Q20 of 98.21% and Q30 of 95.02% (Table S2). A mean of 29,276,016 (97.67%) reads passed quality filters, with 29,171,653 (99.64%) mapping against the avocado var. Hass reference [56] (Table S3). On average, 80.95% of the mapped reads had a minimum depth at a given reference position ≥ 1×, 36.87% ≥ 4×, 3.71% ≥ 10× and 1.02% ≥ 20× (Table S4).

On this combined dataset with the published lcWGS data from Tanzania (95 trees) [51] and the racial controls from the Americas (205 trees) [17], SNP calling retrieved a total of 63,577,355 SNP markers with an average transition/transversion (Ts/Tv) ratio of 2.38 (Table S5), favoring the C:G–T:A transition type over the T:A–C:G transition type (Figure S1). The SNP annotation showed that 41,310,291 (64.98%) and 14,932,366 (23.49%) SNPs, respectively, mapped to non-coding intergenic and intronic regions (Table S6). Among the former, 2,884,776 (4.54%) SNPs fell in the 1 kb window upstream of a transcription start site, 2,433,893 (3.83%) SNPs fell in the 1 kb window downstream of a transcription termination site, and 102,515 (0.16%) SNPs fell in both. On the other hand, a total of 1,901,100 (2.99%) SNPs were exonic, 756,878 (1.19%) synonymous and 1,104,256 (1.74%) nonsynonymous. Among the latter, 35,022 (0.06%) and 4209 (0.01%) SNPs, respectively, caused a gain or loss of a stop codon, and 12,414 (0.02%) altered the splicing pattern. Lastly, for only 735 SNP markers, annotation remained unknown.

**Figure 1:**
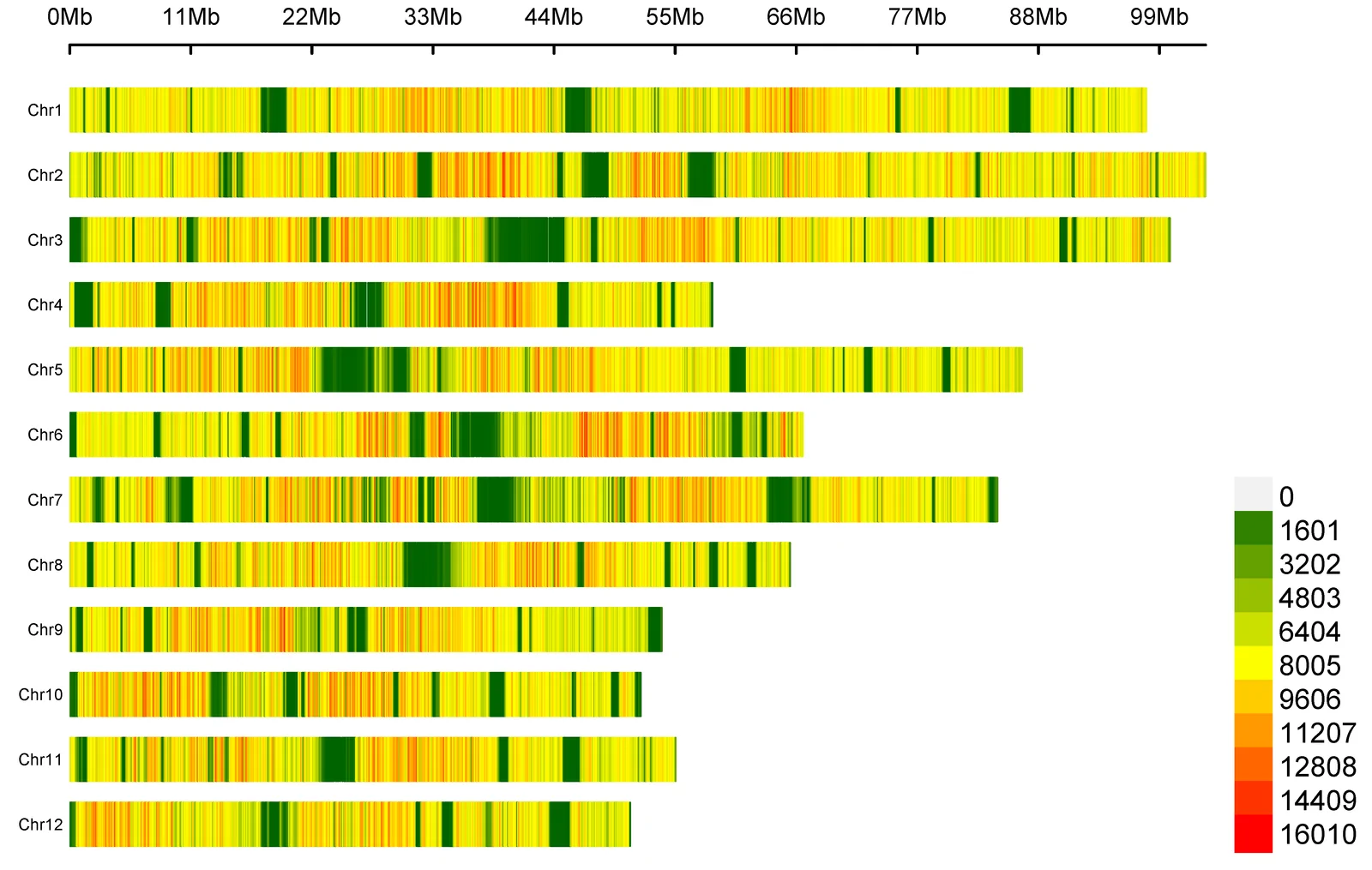
Genome-wide distribution of single nucleotide polymorphisms (SNPs) genotyped using low-coverage whole-genome resequencing (lcWGS) across the avocado (*Persea americana* Mill.) genome from 105 Tanzanian trees sampled in the western, southwestern, and Lake Zone regions of Tanzania. The heatmap shows SNP density along the 12 avocado chromosomes (Chr1–Chr12), calculated using non-overlapping 0.1 Mb genomic windows. Chromosome lengths are displayed according to their physical positions (Mb) on the *x*-axis, which refers to position per chromosome. Colors represent the number of SNPs detected per window, ranging from low density (green) to high density (red), as indicated by the scale bar on the right.

Finally, the density-based visualization of the genome-wide SNP diversity landscape showed extensive yet heterogeneous non-uniformly distributed patterns of polymorphism (Figure 1), likely shaped by avocado’s heterodichogamy [56,57,58]. Localized regions of elevated and reduced SNP variant densities were observed across chromosomes, some of the latter likely coinciding with centromeric regions. This heterogeneity offers an overview of the genomic variation available for the downstream analyses reported in the next subsections for population structure, genetic divergence and ancestry inference among avocado landraces from the Lake Victoria and Tanganyika basins of Tanzania. In addition, as a community resource for other more targeted downstream applications, a subset of 5011 high-quality SNP markers was generated using more stringent marker filtering, reinforcing a minimum sequencing depth of 10× per sample per position, no more than 20% missing data per variant and a minimum allele frequency (maf) of 5%.

### 2.2. Phylogenetic, Multivariate and Admixture Reconstructions

**Figure 2:**
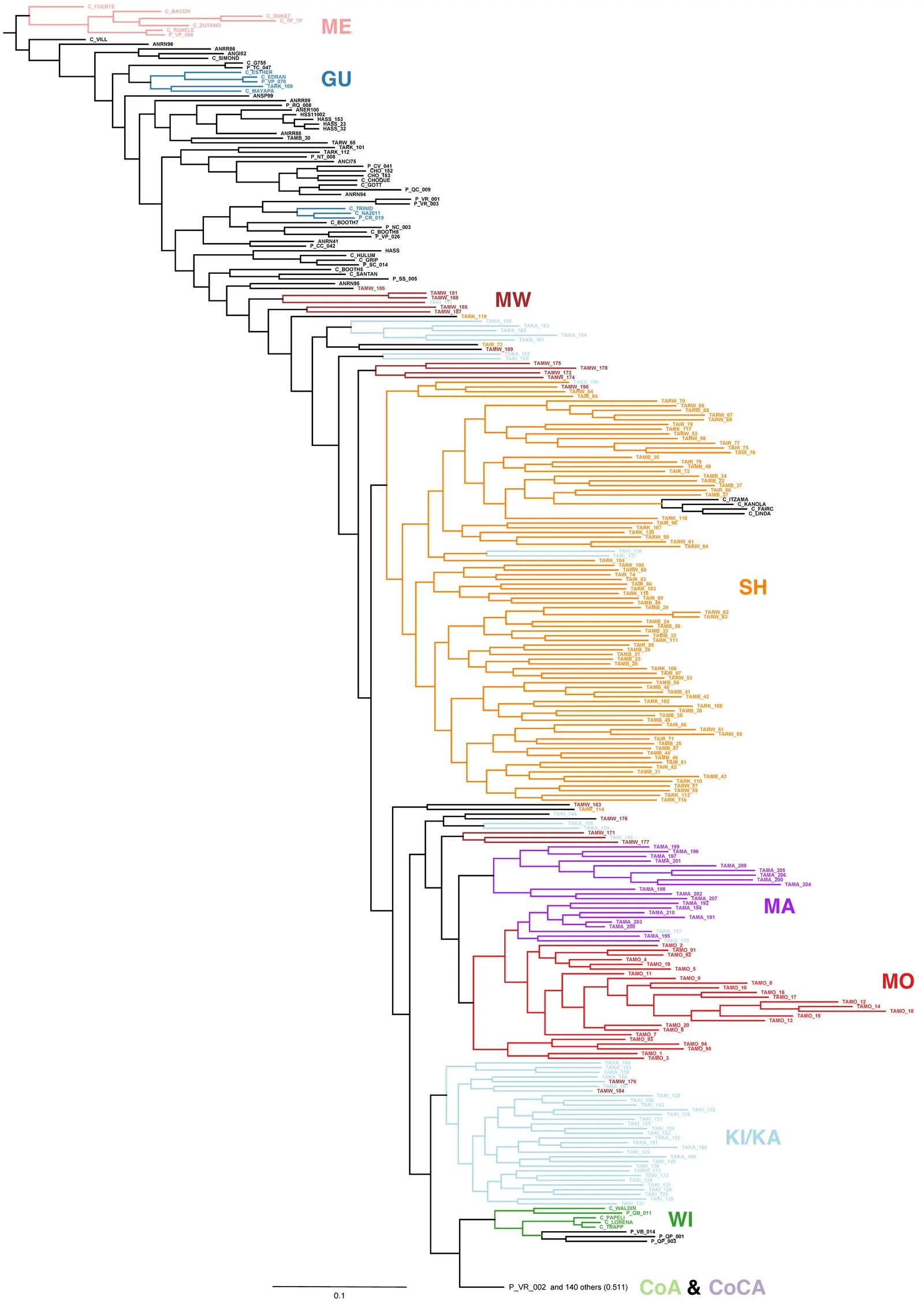
Maximum likelihood phylogeny of Tanzanian avocado germplasm from the Victoria, Tanganyika and Rukwa Lake basins inferred from lcWGS-based SNP markers. The rooted phylogenetic tree was reconstructed using genome-wide SNPs generated by low-coverage whole-genome resequencing (lcWGS) of 105 Tanzanian accessions from the Lake Zone regions of Mara (MA) in dark purple, Mwanza (MW) in dark red and Kagera (KA) in light blue; the Western Zone region of Kigoma (KI), also in light blue; and the Southern Highlands region of Rukwa (name codes ending in RW) in orange. The phylogeny also displays published data for 95 Tanzanian avocado trees from the low- to mid-altitude Morogoro region (MO) in light red and the Southern Highlands (SH, i.e., Iringa, Mbeya and Ruvuma regions) in orange, together with 205 racial-representative reference genotypes of the Mexican (ME in pink), Guatemalan (GU in dark blue), West Indian (WI in dark green), Colombian Andean (CoA in light green) and Caribbean (CoCA in light purple) groups. Branch lengths are proportional to the estimated genetic distance among accessions, while the tree topology illustrates shared ancestry. Branch support values are gathered in Figure S3.

The phylogeny revealed partly monophyletic genomic clustering of Tanzanian germplasm according to provenance geographic origin (Figure 2), with *(i)* Rukwa accessions imbibed within the monophyletic germplasm from the Southern Highlands, *(ii)* tree accessions from Kigoma and Kagera monophyletically grouping as a sister clade of West Indian- and northwest South American-derived lineages, and *(iii)* Mara and Mwanza accessions occupying admixed intermediate phylogenetic positions consistent with rampant inter-racial hybridization and local diversification within the eastern shores of the Lake Victoria Basin (yet also plausibly shaped by incomplete linage sorting, genetic drift and genomic constraints linked with mutation rate variation and effective population size in those regions). These trends are confirmed when inspecting an unrooted version of the same phylogenetic tree but colored by provenance (Figure S2) as well as pairwise *F_ST_* scores (ranging from 0.02 to 0.18, Table S7). Overall, the phylogenetic inference supports the existence of geographically structured genetic divergence in avocados across southern and western Tanzania while highlighting a likely contribution of germplasm exchange and intercrossing in the eastern regions of the Lake Victoria Zone, altogether shaping avocado diversity in the East African Great Lakes region.

The unsupervised dimensionality reduction principal component analysis (PCA) reinforced the genetic differentiation among the major avocado racial control accessions (Table S8), with the recently described northwest South American group [19] splitting at the projection toward the first component (which explained 47.86% of the overall variance) and the West Indian race separating from the other Mesoamerican Mexican and Guatemalan races across the second component (which explained 13.31% of the genomic variance). The PCA analysis also positioned Tanzanian germplasm along a continuum of admixture among the Mesoamerican races, mostly across the second axis of variation. Germplasm from Rukwa exhibited greater affinity with the Southern Highlands, supporting a shared highland genetic background. Meanwhile, accessions from Kigoma and Kagera clustered closer to the West Indian racial control group (Figure S4), whereas Mara and Mwanza occupied more intermingled positions consistent with inter-racial hybridization within the eastern shores of the Lake Victoria Basin. Additional inspection of the third axis of variation (accounting for 8.08% of the genetic variation) against the previous first (Figure S5) and second (Figure S6) axes did not provide further discrimination. The clustering was reinforced when exploring the subset of 5011 high-quality lcWGS-derived SNPs among the first three components (Figures S7–S9, Table S9).

**Figure 3:**
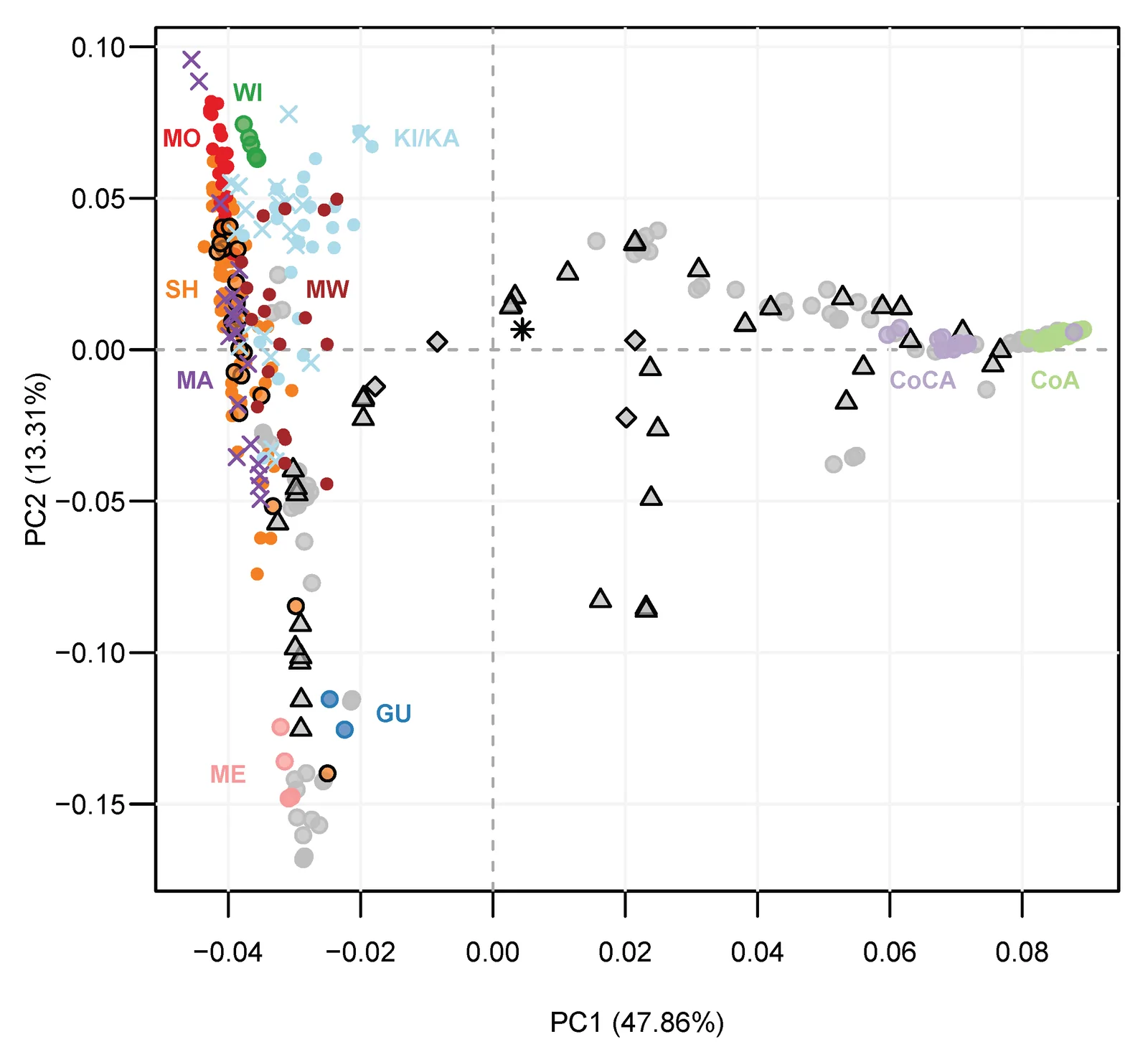
Unsupervised dimensional reduction via principal component analysis (PCA) of Tanzanian avocado germplasm from the Victoria, Tanganyika and Rukwa Lake basins based on lcWGS-derived SNP markers. The analysis included 105 avocado accessions from the Lake Zone regions of Mara (MA), Mwanza (MW) and Kagera (KA), the Western Zone region of Kigoma (KI), and the Southern Highland region of Rukwa (RW), together with published genomic data from 95 Tanzanian avocado trees from the low- to mid-altitude Morogoro region (MO) and the Southern Highlands (SH) regions of Iringa, Mbeya and Ruvuma. The PCA also included 205 racial controls corresponding to the Mexican (ME), Guatemalan (GU), West Indian (WI), Colombian Caribbean (CoCA) and Colombian Andean (CoA) groups. Each point represents an individual genotype, colored according to its regional provenance or reference racial group, as in Figure 2. Gray dots are indicative of double hybrids between the five racial control groups. Triangles, diamonds and the asterisk indicate the triple, tetra- and penta-hybrids, respectively. A detailed assignment of admixed genotypes (i.e., gray dots, triangles and diamonds) to putative parental racial groups is displayed in Figure S4. Principal component (PC)-explained variance is indicated in the axis labels.

Besides the PCA (Figure 3), ADMIXTURE analysis identified progressively finer genetic stratification from *K* = 2 to *K* = 10, with the cross-validation error reaching a plateau at *K* = 9, indicating that nine ancestral components best explained the dataset (Figure S10). At *K* = 2, the dataset was divided into two major genetic groups. At this level, Tanzanian accessions, including those from the Lake Tanganyika, Lake Victoria and Lake Rukwa basins, as well as Morogoro and the Southern Highlands, clustered together with the Guatemalan, Mexican and West Indian racial references, whereas the Colombian Andean and Caribbean controls from northwest South America formed a distinct genetic group. At *K* = 3, additional substructure became evident, with the Guatemalan and Mexican racial controls acquiring purer distinctive profiles. Subsequently, at *K* = 4, the Tanzanian germplasm from the Southern Highlands (including Rukwa provenances) disentangled from the rest, the latter of which exhibited inter-racial admixture with ancestry primarily shared with the West Indian race, a pattern reinforced at *K* = 5, especially for avocados from Kigoma and Kagera. Increasing the number of *a priori* clusters to *K* = 6 revealed finer-scale subdivision within the Tanzanian trees, independently partitioning the Southern Highlands (i.e., Rukwa region included), Kigoma, Kagera and Morogoro provenances from Mwanza and Mara, both of which retained high admixture levels all the way to *K* = 9. At the very same *K* = 6 stratum, northwest South American Colombian Andean trees split from the Caribbean controls. At *K* = 7–8 strata, the Mexican racial controls became purer compared to the Guatemalan controls, and Kigoma/Kagera and Morogoro trees kept singularity.

**Figure 4:**
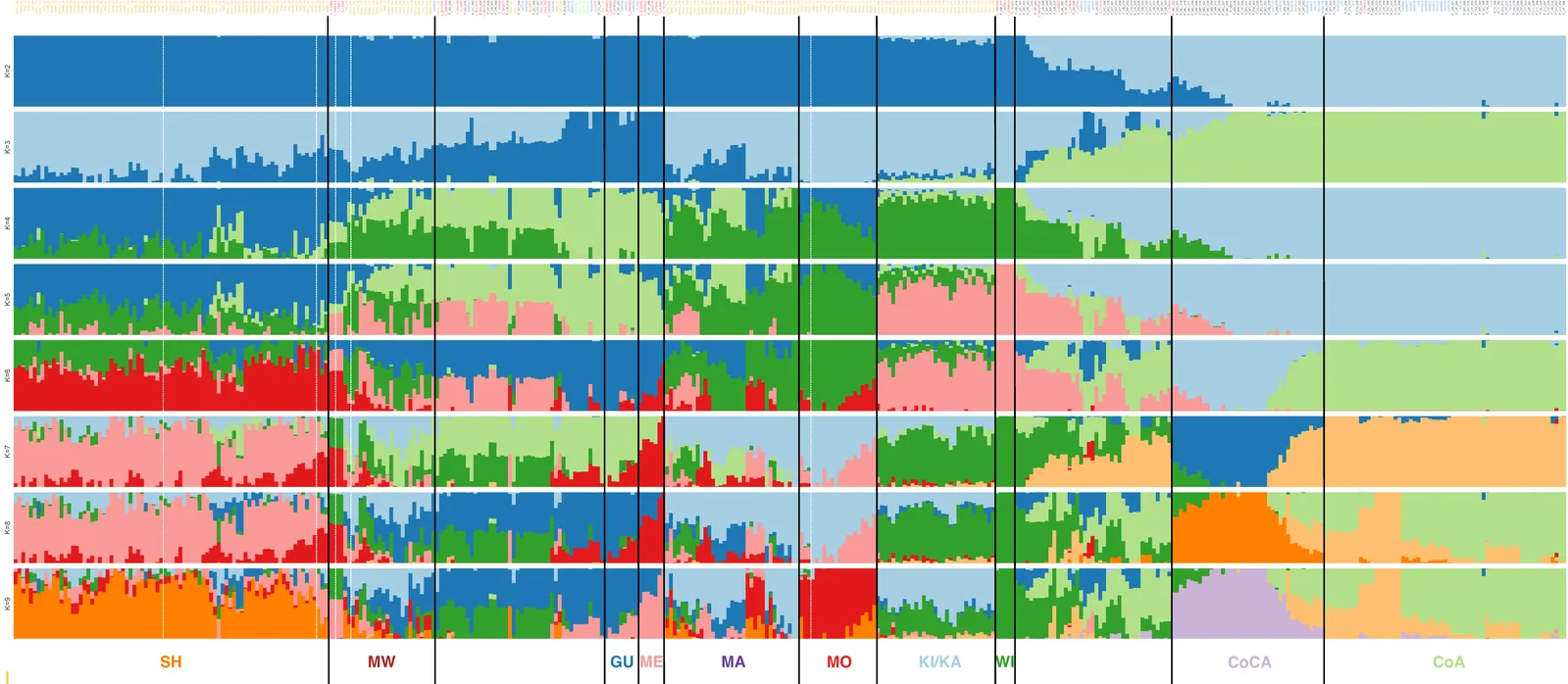
Unsupervised genetic clustering as per ADMIXTURE of Tanzanian avocado germplasm from the Lake Victoria and Tanganyika basins based on genome-wide SNP markers derived from lcWGS. The ADMIXTURE analysis ranged from *K* = 2 to *K* = 9 (at which the cross-validation error stabilized, Figure S6) and covered 105 avocado accessions from the Lake Zone regions of Mara (MA), Mwanza (MW) and Kagera (KA), the Western Zone of Kigoma (KI), and the Southern Highlands region of Rukwa (RW), together with publicly available genomic data from 95 Tanzanian avocado trees from the low- to mid-altitude Morogoro region (MO) and the Southern Highlands (SH) regions of Iringa, Mbeya and Ruvuma. The ADMIXTURE analysis included 205 controls corresponding to the Mexican (ME), Guatemalan (GU), West Indian (WI), Colombian Caribbean (CoCA) and Andean (CoA) groups. Clusters at *K* = 9 are colored as in Figure 2. Codes (as in Table S1) are displayed in the upper row and colored by provenance: in orange, 105 Tanzanian trees from this study and 95 NCBI-available lcWGS data from the Morogoro region and the Southern Highlands; in red, 42 varieties (var. Hass in green); and in light blue, dark blue and black, trees from northwest South America for two subsets and for the entire region, respectively, totalizing 205 racial controls.

Finally, at *K* = 9 (Figure 4), the visualized trends from the phylogenetic and PCA-based unsupervised dimensional reduction, as well as from the previous *K* levels, were corroborated (Table S10). Particularly, Rukwa provenances were entirely embedded within the Southern Highlands clade, Kigoma and Kagera accessions were partly admixed with enrichment for West Indian ancestries, Mwanza and Mara trees harbored signatures of rampant inter-racial hybridization, and avocados from the low- to mid-altitude Morogoro region preserved their purity. In turn, the racial controls were fully resolved in the sense that the Guatemalan, Mexican, West Indian, and Colombian Caribbean and Andean groups maintained distinctive ancestry profiles, validating the ability of the SNP dataset to discriminate among well established avocado racial gene pools.

As another control case, the commercial cultivar ‘Hass’ displayed the expected admixed genetic composition dominated by Guatemalan ancestry with varying Mexican contributions. Overall, this ADMIXTURE analysis indicates that while Tanzanian avocado diversity is dominated by West Indian ancestry with admixed contributions from other Mesoamerican germplasm, it still supports *in situ* purity and local divergence, especially in the Southern Highlands (Rukwa included) and Kigoma/Kagera and Morogoro regions.

## 3. Discussion

The current study offers the first genome-wide assessment of avocado population structure across the Victoria, Tanganyika and Rukwa Lake basins of Tanzania, revealing that the tree species exhibits marked ecogeographic genetic stratification rather than a panmictic distribution. Phylogenetic inference, unsupervised principal component clustering, and ADMIXTURE reconstructions identified three major genomic patterns that broadly support our initial hypotheses: *(i)* a genetic affinity between Rukwa and the previously characterized Southern Highlands germplasm from Iringa, Mbeya and Ruvuma, consistent with a shared highland genetic ancestry; *(ii)* a distinct genetic cluster comprising germplasm from the Kigoma and Kagera regions, characterized by predominant West Indian ancestry; and *(iii)* highly admixed populations in the Mara and Mwanza regions of the Lake Victoria Basin, suggestive of inter-racial hybridization.

These findings insinuate that the genomic landscape of Tanzanian avocado population stratification may have been shaped by the combined influence of environmental heterogeneity, localized micro-evolutionary processes, and historical germplasm movement across the East African Great Lakes region. These patterns offer a framework for interpreting the ecological and historical drivers underlying avocado diversification in the Western Rift and Lake Zone, enabling the conservation and utilization of locally adapted genetic resources.

### 3.1. Rukwa and the Southern Highlands Genetic Corridor

Genomic diversity of avocado across Tanzania’s Southern Highlands, Western Zone and Lake Zone regions reveals a possible interplay between topographical barriers, environmental adaptation, and anthropogenic dispersal of seed planting material. Our results unveil genetic stratification that aligns with the country’s diverse agroecological zones and historical and contemporary routes of planting material exchange. For example, the genetic affinity between germplasm from Rukwa and the previously characterized Southern Highlands germplasm from Iringa, Mbeya and Ruvuma may suggest shared genetic connectivity and possible historical and contemporary exchange across these bridged highland regions [49,50]. Geographically, Rukwa is part of the Southern Highlands and shares highland agroecological characteristics with regions such as Mbeya and Njombe. This highland corridor is characterized by cooler temperatures and well-distributed rainfall, favoring the survival of Mexican/Guatemalan cold-tolerant genotypes [51]. This similarity suggests that the avocado germplasm in Rukwa is genetically consistent with the broader Southern Highlands germplasm, potentially reflecting regional exchange of planting material along trade routes such as the Sumbawanga–Mbeya–Iringa Road [6] and a shared farmer preference or ecological filtering for highland-adapted cultivars [59].

### 3.2. Distinct Avocado Racial Ancestry in Kigoma and Kagera Regions

Meanwhile, the distinct genomic signatures in avocados from Kigoma and Kagera point toward a distinct introduction and eco-evolutionary history. While geographically associated with different lake basins, with Kigoma in the Lake Tanganyika Basin and Kagera in the Lake Victoria Basin, both regions are connected to the broader Albertine Rift–Great Lakes landscape and share historical links with neighboring countries, including Burundi, Rwanda, Uganda, and the Democratic Republic of Congo (DRC). From a historical point of view, agricultural extension during the colonial and post-colonial eras in these neighboring countries may have prioritized, through transboundary gene flow, lowland-adapted West Indian ancestries together with some mid-altitude provenances, the latter for their superior oil content and transportability [60]. A cradle for this intermingling may have been the historical port of Kigoma, which has served as a primary maritime link between Tanzania, Burundi and the DRC (i.e., the Lake Tanganyika–Burundi–DRC axis). The movement of planting material and people along these lake-based trade networks may have facilitated the introduction of diverse avocado germplasm into Kigoma. This could explain the presence of lowland-adapted West Indian genotypes, with proximity to the control group from northwestern South America [19], mirroring those cultivated in other regions of Central America [61], such as Nicaragua [25]. Similarly, the Kagera region forms part of the Lake Victoria Basin and serves as a terrestrial bridge to Uganda and Rwanda. In these neighboring countries, early 20th-century agricultural introductions may have also emphasized mid-altitude-adapted provenances, without disregarding cold-tolerant varieties due to the local topographic heterogeneity. The continuous flow of people and goods across these porous borders likely facilitated a genetic spillover and swamping, hypothesizing high genomic similarity between Kagera’s avocado populations and those of the broader East African Montane ecosystem.

Ecological divergent selection may have also shaped this ancestry [62]. In particular, the steep altitudinal gradients of the Western Rift Valley could have imposed directional and divergent selection pressures on avocado populations. In this sense, a dominance of West Indian ancestry in the region, more toward the northwest South American clade [19] than observed in the other Tanzanian provenances examined so far [51], may reflect allelic filtering shaped by the physiological adaptation thresholds [63] to the lowland [64] and mid-altitude [65] climates found in the Tanganyika lake shores of Kigoma and the slopes of Kagera, the latter on the west of Lake Victoria.

### 3.3. Genetic Divergence in the Lake Victoria Basin Across the Mara and Mwanza Clades

The clustering of avocados from Mara and Mwanza as genetically intermediate and intercrossed clades may imply a process of inter-racial hybridization and *in situ* ecology-driven diversification [9,66] since, unlike the highland-dominated Southern Highlands [50,51], the Lake Victoria Basin offers a more stable, humid, and warmer lacustrine climate [67]. The intermediate genetic position of these populations could suggest an inter-racial hybridization and introgression dynamic [68], where early introductions (likely of West Indian origin via historical coastal-to-inland trade routes) have intercrossed with subsequent introductions from the Tanzanian highlands and neighboring countries [66], matching agroecological niches only achieved by intermixed genotypes. Specifically, as in the previous case, the northern Mara region’s genetic uniqueness may be influenced by its proximity to Kenya and the commercially active village of Tarime. Trade through Lake Victoria and across the Sirari border has likely introduced landraces throughout the north of Tanzania [69], with a possible distinct history of farmer selection and ecological adaptation [70]. The intermediate genetic position of Mara and Mwanza populations may so be the result of hybridization between these northern introductions and older coastal-to-inland Antillean varieties (e.g., the Morogoro West Indian type) that were introduced during the colonial period via central trade caravan routes.

Genetic isolation could have also been a major driving force in the uniqueness of the Mara/Mwanza clade. Specifically, the vast expanse of Lake Victoria to the north and the Serengeti plains to the east may have created a narrow north–south corridor suitable for avocado cultivation, with a moderate degree of geographic and ecological isolation for these populations. In turn, *ad hoc* hypotheses to be further considered include incomplete lineage sorting and random genetic drift, both of which could as well have allowed for the fixation of admixed alleles, leading to distinct clades genetically divergent from the purer ancestries found in the Southern Highlands or the Western Rift.

### 3.4. Perspectives

The three major geographic clustering patterns described in the sections above illustrate that the western and lacustrine regions of Tanzania do not exist as genetic islands but rather as potential receptive sinks for avocado germplasm from the entire Great Lakes region, creating a unique melting pot of avocado diversity in East Africa. These results are paramount for future breeding and management programs [71,72,73] in the region. Specifically, the unique outcrossing identity of the Mwanza/Mara clades may offer candidate germplasm for future evaluation. Meanwhile, the Kigoma/Kagera populations could serve as a resource for both lowland- and mid-altitude adaptation genes. Eventually, recognizing avocado populations from these regions as a source of novel distinct germplasm would be essential for the systematic conservation of Tanzania’s avocado diversity and its omics-assisted utilization [74,75,76,77,78,79], management [80,81,82,83,84,85] and propagation [86,87] as rootstock saplings [88,89,90,91] or varietal trees and scions [92] in the face of pressing land use [67], climate resilience [93,94,95] and sustainability [96] demands.

## 4. Materials and Methods

### 4.1. Tree Sampling

As part of this study, 105 avocado adult elite ‘plus trees’ were identified and monitored during August/September 2024 in the southwestern region of Rukwa (Sumbawanga and Kalambo district: 1747–1986 m a.s.l., *n* = 20), the western region of Kigoma (Buhingwe, Kasulu, Kibondo and Kakonko districts: 1208–1641 m a.s.l., *n* = 25) along the eastern shore of Lake Tanganyika, and in the Lake Zone regions of Kagera (Ngara district: 1449–1800 m a.s.l., *n* = 23), Mwanza (Sengerema and Ilemela districts: 1211–1251 m a.s.l., *n* = 18) and Mara (Tarime district: 1555–1596 m a.s.l., *n* = 19) along the western, southern, and eastern shores of Lake Victoria, respectively (Figure S11, Table S1). The complete tree inventory comprised five regions, ten districts, 15 wards and 17 villages distributed across southwestern, western and northwestern Tanzania (Table 1).

Global positioning system (GPS) georeferencing and quantitative measurements (i.e., tree height and trunk diameter) were recorded *in situ* for each tree. Additionally, five young leaves were sampled in duplicate for each tree for DNA extraction. Leaf tissue was dried and preserved within porous tea bags placed in hermetic plastic boxes with color-indicating silica gel (Sigma Aldrich, Hamburg, Germany).

### 4.2. Genomic DNA Extraction and Low-Coverage Whole-Genome Sequencing (lcWGS)

Total genomic DNA was extracted from the 105 leaf samples using a DNeasy Plant Mini Kit (QIAGEN, Hilden, Germany). DNA was quantified on Agarose gels and with a NanoDrop^®^ 2000 spectrophotometer (Thermo Fisher, Waltham, MA, USA), as per the OD260/OD280 ratio (1.8–2.0, >0.5 µg). As a second step, an Illumina TruSeq™ Nano DNA Sample Prep Kit was used to prepare genomic libraries, which were then 150 bp pair-end sequenced on an Illumina Novaseq at CD Genomics (Shirley, NJ, USA).

### 4.3. Sequencing Data Processing

CASAVA software v.1.6 (Illumina Inc., San Diego, CA, USA) was used for base calling. Resultant FASTQ files were trimmed for 3′ adapter contamination. In addition, low-quality reads were removed in Fastp v.0.20.0. if Q scores were <20 in a 5 bp 3′–5′ sliding window. Paired sequences were also discarded when the number of N bases was ≥5 or their length was <50 bp. At this step, sequence data for the 105 trees was merged with the FASTQ files for avocado trees already surveyed in the Tanzanian Morogoro and Southern Highlands regions of Iringa, Mbeya and Ruvuma (NCBI BioProject ID PRJNA1442766) [51], as well as with the FASTQ files of 205 racial-informative avocados from the Americas (NCBI BioProject ID PRJNA1311704) [17], spanning 42 commercial cultivars, the Mesoamerican races and the recently reported Andean and Caribbean groups from northwest South America [19]. All reads were handled under the exact same pipeline.

Filtered reads were then mapped, using BWA v.0.7.12-r1039, to Queensland’s reference genome assembly of avocado cv. Hass (downloadable from the AvoBase repository at https://www.avocado.uma.es/easy_gdb/downloads.php, accessed on 3 August 2026) [56]. Mapped file transformation from SAM to BAM formats was done in Picard v.1.107 (https://broadinstitute.github.io/picard/, accessed on 3 August 2026) with the commands FixMateInformation for paired-end consistency and MarkDuplicates for duplicate removal. SNP variant calling was then performed using the HaplotypeCaller tool in GATK software v.4.5.0.0 [97], generating per-sample GVCF files that were subsequently genotyped and combined across samples and chromosomes using GATK’s joint genotyping GenotypeGVCFs, CombineGVCFs and GatherVcfs commands, respectively. The SelectVariants operator of the same software was then utilized for SNP marker extraction from the merged GVCF file.

Quality filtering of SNP markers was performed using the VariantFiltration command of the same GATK pipeline. Markers were retained only if Fisher’s test of strand bias (FS) ≤ 60, mapping quality (MQ) ≥ 40, quality depth (QD) ≥ 2, ReadPosRankSum ≥ −8.0 and MQRankSum > −12.5. Annotation of the resulting SNP marker dataset was performed using ANNOVAR software v.4.0 [98]. Finally, as a more manageable downstream result for the community, an additional high-coverage SNP panel was retrieved with VCFtools v.2015 [99] by retaining markers with a minimum sequence depth (SD) of 10× per sample, genotypes present in at least 80% of the compiled samples (i.e., missing data per variant ≤ 20%) and a minimum minor allele frequency (maf) of 5%. This high-quality dataset intrinsically reduces molecular marker pseudo-replication and linkage disequilibrium (LD), which may arise from high SNP density and the resultant extensive among-marker LD within haplotype blocks. Therefore, the marker subset configures itself as a more downloadable, shareable, traceable and utilizable resource for upcoming population genomics (e.g., genome-wide selection scans, GWSS), genetic mapping (e.g., genome-wide association studies, GWAS) and molecular breeding analyses (e.g., genomic prediction and genomic forecasting), among other valuable last-generation approaches.

### 4.4. Population Genomics Analysis

Phylogenetic tree inference on the full dataset was done using the Maximum Likelihood algorithm with Shimodaira–Hasegawa node support, as implemented in the FastTree 2.2 plugin (http://www.microbesonline.org/fasttree/, accessed on 3 August 2026) of Geneious v.2026.0.2 software [100]. Unsupervised dimensional reduction via principal component analysis (PCA) was carried out for the full and the high-quality SNP sets with GCTA software v.1.93.3 (https://github.com/jianyangqt/gcta, accessed on 3 August 2026). Computation of *F_ST_* scores and PCA visualization were done with customized R scripts. Lastly, unsupervised genetic admixture inference was done with ADMIXTURE software v.1.3.1 (http://dalexander.github.io/admixture, accessed on 3 August 2026) for values of *K* ranging from 2 to 10. Cross-validation (CV) error scores were used to determine the optimal *K* value. All software handled missing genotypes natively.

## 5. Conclusions

The landscape of genomic divergence in avocado populations from the southwestern, western and Lake Zone regions of Tanzania reflects the country’s complex biogeographic, historical, and contemporary patterns of germplasm exchange. This study demonstrates that genetic diversity in the studied populations is not randomly distributed as expected under a panmictic model, instead following a trend in which: *(i)* a proximity between germplasm from Rukwa and previously characterized Southern Highlands germplasm is likely driven by shared highland agroecological filtering and possible historical and domestic exchange of planting material, *(ii)* a predominant West Indian genetic ancestry in the Western Rift regions of Kigoma and Kagera may result from historical and contemporary transboundary germplasm exchange with neighboring Great Lakes countries, including Burundi, Rwanda and Uganda, and *(iii)* the emergence of genetically intermediate and highly admixed populations in Mwanza and Mara could suggest a history of inter-racial hybridization and possible *in situ* local selection within the Lake Victoria Basin, without disregarding other underlying driving processes such as incomplete lineage sorting and genetic drift. Yet, as far as the study goes, it demonstrates that patterns of genomic stratification may reflect geographical gradients while not necessarily proving that the latter caused the former. In order to fully unveil the causation process underneath this pattern of genomic and geographical concordance, upcoming studies should aim to disentangle *ad hoc* hypotheses concerning isolation by distance, isolation by ecology and isolation by trading preferences, all of which are, in turn, known to covary with ecogeographical gradients in the region. These future studies must also ensure higher sequencing coverage and supporting phenotyping characterizations. Overall, these findings highlight the southwestern, western and Lake Zone regions of Tanzania as a major secondary center of avocado diversity in East Africa. The presence of genetically distinct and admixed landraces, particularly in the Lake Victoria and Lake Tanganyika basins, reinforces the need for targeted *in situ* conservation, as well as for the integration of these resilient local genotypes into future rootstock pre-breeding and selection programs. Ultimately, understanding these genomic patterns of divergence is vital for securing and diversifying avocado production in the face of the climatic and market volatilities in East Africa.

## Figures and Tables

**Table 1:** Provenances of the 105 avocado elite ‘plus trees’ from the Western Zone region of Kigoma, the Southern Highlands region of Rukwa, and the Lake Zone regions of Kagera, Mwanza and Mara.

Region	District	Ward	Village	Number of Samples	Sampling Site Elevation (m a.s.l.)
Rukwa	Sumbawanga	Mollo	Isesa	7	1868–1904
Rukwa	Sumbawanga	Katandala	NA	6	1794–1806
Rukwa	Sumbawanga	Asilia	NA	1	1750
Rukwa	Sumbawanga	NA	Katwa	2	1747
Rukwa	Sumbawanga	NA	NA	2	1804–1806
Rukwa	Kalambo	Lyowa	Charamimwe	2	1904-1986
Kigoma	Buhingwe	Buhingwe	Buhingwe	3	1369–1375
Kigoma	Buhingwe	Buhingwe	Murela	9	1369–1385
Kigoma	Kasulu	Kasange	Heru juu	9	1619–1637
Kigoma	Kasulu	Heru juu	Kasunga	1	1641
Kigoma	Kibondo	Mabamba	Mabamba	1	1279
Kigoma	Kakonko	Kiziguzigu	Kabingo	2	1208
Kagera	Ngara	Kabanga	Kabanga	8	1449–1800
Kagera	Ngara	Shibimba	Shibimba	1	1712
Kagera	Ngara	Ngara Mjini	Kibimba	13	1659–1719
Kagera	Ngara	Ngara Mjini	Mukididili	1	1662
Mwanza	Sengerema	Bukokwa	Mwamanyiri	9	1211–1216
Mwanza	Ilemela	Nyasaka	Nyasaka Centre	9	1232–1251
Mara	Tarima	Kitare	Nyamasamore	8	1555–1575
Mara	Tarime	Kitare	Kibumaye	11	1565–1596

## Data Availability

Downstream data is available in this article and in the Supplementary Materials. The 105 FASTQ data presented in this study is openly available in NCBI BioProject at https://www.ncbi.nlm.nih.gov/bioproject/PRJNA1534007 (accessed on 22 October 2026); reference number PRJNA1534007.

## Supplementary Materials

The following supporting information can be downloaded at https://www.mdpi.com/article/10.3390/ijms27219699/s1. **Table S1**. Provenance data of 105 Tanzanian avocado trees sampled from the Lake Zone regions of Mara, Mwanza and Kagera, the western region of Kigoma, and the southwestern region of Rukwa. **Table S2**. Overall statistics of sequencing data including number of reads, total number of bases (bp) and GC percentage for each of the 105 samples enlisted above. **Table S3**. Statistics of mapping rate including total number of reads (i.e., total reads), number of reads aligned to the reference genome including single-end and paired-end alignments (i.e., mapped reads), and percentage of reads aligned to the reference genome relative to the total number of reads (i.e., mapping rate). **Table S4**. Statistics of coverage at 1×, 4×, 10× and 20× mean minimum depth for each of the 105 samples. **Table S5**. Statistics of SNP calling for each of the 105 samples including number of homozygous genotypes consistent with the reference genome (HOM_REF), number of heterozygous genotypes (HET), number of homozygous genotypes inconsistent with the reference genome (HOM_ALT), number of transitions (*Ts*, substitutions between purines and purines, or pyrimidines and pyrimidines), number of transversions (*Tv*, substitutions between purines and pyrimidines) and ratio of transitions to transversions (*Ts*/*Tv*). **Table S6**. Summary of the SNP annotation statistics (i.e., counts and percentage), discretizing among markers within exonic regions, synonymous and nonsynonymous substitutions, variants causing a gain (stopgain) or a loss (stoploss) of a stop codon, SNPs within 2 bp of a splicing junction (splicing), markers within intronic and intergenic regions, as well as those 1 kb upstream of a transcription start site (upstream), 1 kb downstream of a transcription termination site (downstream), or both. **Table S7**. Pairwise FST scores among the newly screened trees from the Lake Zone regions of Mara (MA), Mwanza (MW) and Kagera (KA), and the western region of Kigoma (KI) and the southwestern region of Rukwa (RK), together with NCBI-available Tanzanian avocado trees from the Southern Highlands (SH) of Iringa, Mbeya and Ruvuma regions. **Table S8**. Three main principal components from an unsupervised dimensionality-reduction genetic PCA across the 105 Tanzanian avocado tree samples genotyped with all lcWGS-derived SNP markers as part of this study. **Table S9**. Three main principal components from an unsupervised dimensionality-reduction genetic PCA based on the subset of 5011 high-quality lcWGS-derived SNP markers (i.e., filtered at minimum sequencing depth of 10× per sample per position, no more than 20% missing data per variant and *maf* of 5%), retrieved from the 105 Tanzanian avocado trees genotyped as part of this study. **Table S10**. *Q* matrix from the ADMIXTURE-based unsupervised genetic clustering (*K* = 9) across the lcWGS-genotypes 105 avocado trees. **Figure S1**. Barplot diagram indicating the frequency of the six possible nucleotide substitution classes, grouped into transitions (*Ts*) and transversions (*Tv*), the *x*-axis indicating the total number of mutations for each substitution class and illustrating the predominance of transition mutations over transversions. **Figure S2**. Unrooted FastTree-based phylogenetic tree colored by provenance utilizing lcWGS-derived SNPs screened across the 105 Tanzanian avocado trees from the Lake Zone regions of Mara, Mwanza and Kagera, and the western region of Kigoma and the southwestern region of Rukwa, together with 95 NCBI-available Tanzanian avocado trees from the low- to mid-altitude Morogoro region and the Southern Highlands of Iringa, Mbeya and Ruvuma regions (all in purple), in addition of 205 published racial controls including 42 commercial varieties (blue branches, *var.* Hass in red) and trees from northwest South America (orange, yellow and green branches for two subsets and for the entire region, respectively). **Figure S3**. Maximum likelihood rooted phylogeny with branch support inferred from lcWGS-based SNP genotyping. Germplasm spans 105 newly screened Tanzanian avocado accessions from the Lake Zone regions of Mara (MA) in dark purple, Mwanza (MW) in dark red and Kagera (KA) in light blue, the Western Zone region of Kigoma (KI) also in light blue, and the Southern Highlands region of Rukwa (name codes ending in RW) in orange, together with published data from 95 Tanzanian avocado trees from the low- to mid-altitude Morogoro region (MO) in light red and the Southern Highlands (SH, i.e., Iringa, Mbeya and Ruvuma regions) in orange, as well as 205 racial-representative reference genotypes of the Mexican (ME in pink), Guatemalan (GU in dark blue), West Indian (WI in dark green), and Colombian Andean (CoA in light green) and Caribbean (CoCA in light purple) groups. **Figure S4**. Unsupervised dimensionality-reduction genetic PCA of Tanzanian avocado germplasm from the Victoria, Tanganyika and Rukwa Lake basins based on all lcWGS-derived SNP markers. The analysis includes 105 avocado accessions from the Lake Zone regions of Mara (MA), Mwanza (MW) and Kagera (KA), the western region of Kigoma (KI), and the southwestern region of Rukwa (RW), together with published genomic data from 95 Tanzanian avocado trees from the low- to mid-altitude Morogoro region (MO), and the Southern Highlands (SH) regions of Iringa, Mbeya and Ruvuma. The PCA also includes 205 racial controls corresponding to the Mexican (ME), Guatemalan (GU), West Indian (WI), Colombian Caribbean (CoCA) and Colombian Andean (CoA) groups. Each point represents an individual genotype, colored according to its regional provenance or reference racial group as in Figure 2. The remaining dots mark double hybrids between these last five groups, the interior and the edge colored according to the putative parental groups. Triangles mark tripe hybrids, the interior and the edge colored according to two of the putative parental groups, and its direction pointing towards the third contributing group. Diamonds mark tetra hybrids, the interior and the edge colored according to the two most distant putative parental groups, while the other two groups can be resolved without ambiguity based on the proximity to the five racial control groups. Finally, the asterisk marks the penta-hybrid among all five main groups (i.e., Mexican race, Guatemalan race, West Indian race, and Caribbean and Andean groups from northwest South America). **Figure S5**. Unsupervised genetic clustering via principal component analysis emphasizing the first and the third principal components (PC) in 105 Tanzanian avocado trees from the western and Lake Zone regions, together with 95 published Tanzanian avocado trees from the Morogoro region and the Southern Highlands, and 205 NCBI-available racial controls, all screened with the full lcWGS-derived SNP marker set (coloring pattern and dot shape as in Figure S4). **Figure S6**. Unsupervised genetic clustering via principal component analysis emphasizing the second and the third principal components (PC) in 105 Tanzanian avocado trees from the western, southwestern and Lake Zone regions, together with 95 published Tanzanian avocado trees from the Morogoro region and the Southern Highlands, and 205 NCBI-available racial controls, all screened with the full lcWGS-derived SNP marker set (coloring pattern and dot shape as in Figure S4). **Figure S7**. Unsupervised dimensionality-reduction genetic PCA of Tanzanian avocado germplasm from the Victoria, Tanganyika and Rukwa Lake basins based on the subset of 5011 high-quality lcWGS-derived SNP markers. This marker subset aims controlling for molecular marker pseudo-replication and LD that could arise from elevated SNP density and the extensive among-marker haplotype blocks. The analysis includes 105 avocado accessions from the Lake Zone regions of Mara (MA), Mwanza (MW) and Kagera (KA), the western region of Kigoma (KI), and the southwestern region of Rukwa (RW), together with published genomic data from 95 Tanzanian avocado trees from the low- to mid-altitude Morogoro region (MO), and the Southern Highlands (SH) regions of Iringa, Mbeya and Ruvuma. The PCA also includes 205 racial controls corresponding to the Mexican (ME), Guatemalan (GU), West Indian (WI), Colombian Caribbean (CoCA) and Colombian Andean (CoA) groups. The high-quality lcWGS-derived SNP markers were gathered across the compilation of all sample panels using stringent marker filtering (*i.e.*, minimum sequencing depth of 10× per sample per position, no more than 20% missing data per variant and *maf* of 5%). Each point represents an individual genotype, colored according to its regional provenance or reference racial group as in Figure 2. The remaining dots mark double hybrids between these last five groups, the interior and the edge colored according to the putative parental groups. Triangles mark tripe hybrids, the interior and the edge colored according to two of the putative parental groups, and its direction pointing towards the third contributing group. Diamonds mark tetra hybrids, the interior and the edge colored according to the two most distant putative parental groups, while the other two groups can be resolved without ambiguity based on the proximity to the five racial control groups. Finally, the asterisk marks the penta-hybrid among all five main groups (i.e., Mexican race, Guatemalan race, West Indian race, and Caribbean and Andean groups from northwest South America). **Figure S8**. Unsupervised genetic clustering via principal component analysis emphasizing the first and the third principal components (PC) in 105 Tanzanian avocado trees from the western and Lake Zone regions, together with 95 published Tanzanian avocado trees from the Morogoro region and the Southern Highlands, and 205 NCBI-available racial controls, all screened with the subset of 5011 high-quality lcWGS-derived SNP markers (coloring pattern and dot shape as in Figure S4). **Figure S9**. Unsupervised genetic clustering via principal component analysis emphasizing the second and the third principal components (PC) in 105 Tanzanian avocado trees from the western, southwestern and Lake Zone regions, together with 95 published Tanzanian avocado trees from the Morogoro region and the Southern Highlands, and 205 NCBI-available racial controls, all screened with the subset of 5011 high-quality lcWGS-derived SNP markers (coloring pattern and dot shape as in Figure S4). **Figure S10**. Cross-validation (CV) error from the admixture-based unsupervised genetic clustering (from *K* = 2 to *K* = 10) from the western and Lake Zone regions, together with 95 published Tanzanian avocado trees from the Morogoro region and the Southern Highlands, and 205 NCBI-available racial controls, all screened with lcWGS-derived SNP markers. The ADMIXTURE analysis utilized a block relaxation algorithm starting with a default random seed of 43 (replacing it for each of the ten independent runs per *K*), a 5-fold cross-validation and a convergence tolerance of Δ < 0.0001. **Figure S11**. Map of Tanzania depicting sampling points of the 105 newly screened avocado trees from the Lake Zone regions of Mara, Mwanza and Kagera, and the western region of Kigoma and the southwestern region of Rukwa, together with 95 additional NCBI-available trees from the low- to mid-altitude Morogoro region and the Southern Highlands of Iringa, Mbeya and Ruvuma regions.
