# Supplementary material for: Avocado (Persea americana Mill.) Genomic Divergence Across the Victoria, Tanganyika and Rukwa Lake Basins of Tanzania
Source: Int J Mol Sci. 2026 Oct 29;27(21):9699. doi: 10.3390/ijms27219699 (PMC13763250; doi:10.3390/ijms27219699)
Supplement: Supplementary file 1 [file ijms-27-09699-s001.zip › ijms-4614099-supplementary/FigS1.pdf]

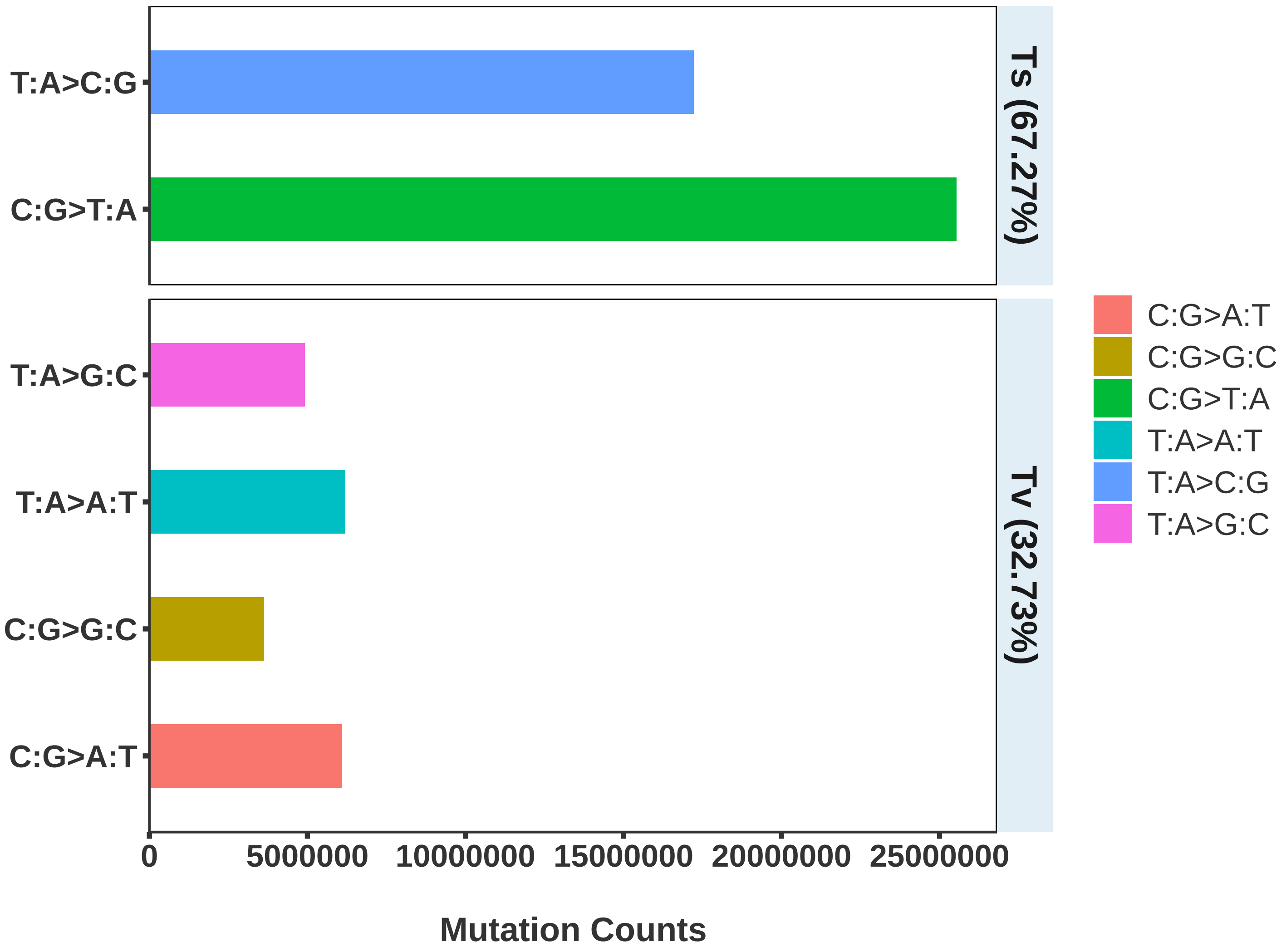

Ts (67.27%)
Tv (32.73%)
T:A>C:G
C:G>T:A
T:A>G:C
T:A>A:T
C:G>G:C
C:G>A:T
0
5000000
10000000
15000000
20000000
25000000
Mutation Counts
C:G>A:T
C:G>G:C
C:G>T:A
T:A>A:T
T:A>C:G
T:A>G:C
