# Supplementary material for: Avocado (Persea americana Mill.) Genomic Divergence Across the Victoria, Tanganyika and Rukwa Lake Basins of Tanzania
Source: Int J Mol Sci. 2026 Oct 29;27(21):9699. doi: 10.3390/ijms27219699 (PMC13763250; doi:10.3390/ijms27219699)
Supplement: Supplementary file 1 [file ijms-27-09699-s001.zip › ijms-4614099-supplementary/FigS3.pdf]

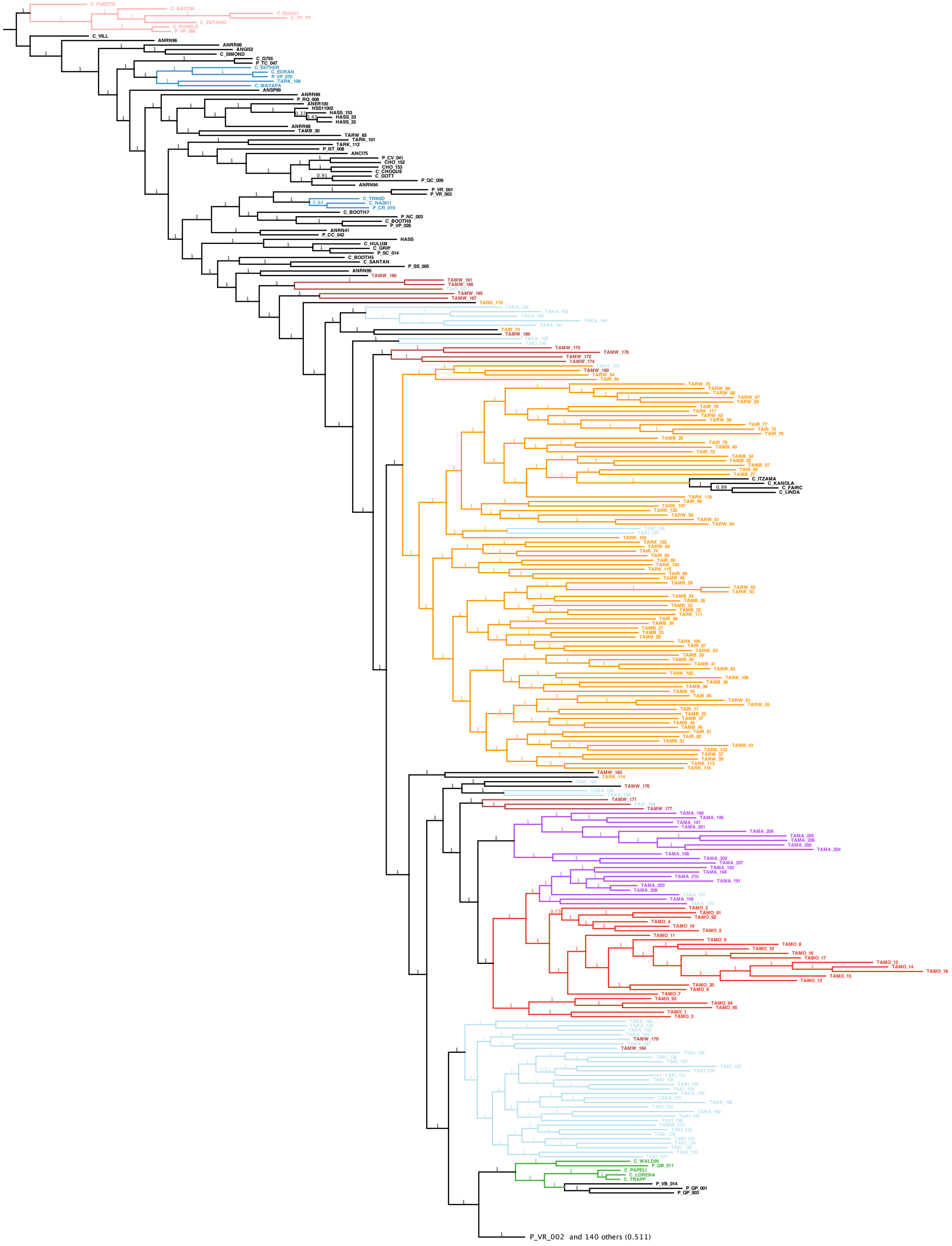
C_FUERTE
C_BACON
C_DUKE7
C_TP_TP
C_ZUTANO
C_RUHELE
P_VP_066
C_VILL
ANRN96
ANRR86
ANGI52
C_SIMOND
C_G755
P_TC_047
C_ESTHER
C_EDRAN
P_VP_070
TARK_109
C_MAYAPA
ANSP99
ANRR89
P_RQ_008
ANER100
HSS11002
HASS_153
HASS_23
HASS_32
ANRR88
TAMB_30
TARW_65
TARK_101
TARK_112
P_NT_008
ANCI75
P_CV_041
CHO_152
CHO_153
C_CHOQUE
C_GOTT
P_QC_009
ANRN94
P_VR_001
P_VR_003
C_TRINID
C_NA2011
P_CR_019
C_BOOTH7
P_NC_003
C_BOOTH8
P_VP_026
ANRN41
P_CC_042
HASS
C_HULUM
C_GRIP
P_SC_014
C_BOOTH5
C_SANTAN
P_SS_005
ANRN95
TAMW_186
TAMW_181
TAMW_188
TAMW_185
TAMW_187
TARK_119
TAIR_73
TAMW_189
TAMW_175
TAMW_178
TAMW_172
TAMW_174
TAMW_190
TARW_54
TAIR_84
TARW_70
TARW_66
TARW_68
TARW_67
TARW_69
TAIR_78
TARK_117
TARW_82
TARW_56
TARW_77
TAIR_75
TAIR_76
TAMB_35
TAIR_79
TAMB_49
TAIR_72
TAMB_34
TAMB_22
TAMB_37
TAIR_86
TAMB_27
C_ITZAMA
C_KANOLA
C_FAIRC
C_LINDA
TARK_118
TAIR_90
TARK_107
TARK_120
TARW_58
TARW_61
TARW_64
TARK_104
TARK_105
TARW_60
TAIR_74
TAIR_83
TAIR_80
TARK_103
TARK_115
TAIR_89
TAMB_48
TAMB_29
TARW_62
TARW_63
TAMB_24
TAMB_26
TAMB_33
TAMB_32
TARK_111
TAIR_88
TAMB_39
TAMB_21
TAMB_23
TAMB_28
TARK_106
TAIR_87
TARW_53
TAMB_50
TAMB_40
TAMB_41
TAMB_42
TARK_102
TARK_108
TAMB_36
TAMB_38
TAMB_45
TAIR_85
TARW_51
TARW_55
TAIR_71
TAMB_25
TAMB_47
TAMB_44
TAMB_46
TAIR_81
TAIR_82
TAMB_31
TAMB_43
TARK_110
TARW_57
TARW_59
TARK_113
TARK_116
TAMW_183
TARK_114
TAMW_176
TAMW_171
TAMW_177
TAMA_199
TAMA_196
TAMA_197
TAMA_201
TAMA_209
TAMA_205
TAMA_206
TAMA_200
TAMA_204
TAMA_198
TAMA_202
TAMA_207
TAMA_192
TAMA_194
TAMA_210
TAMA_191
TAMA_203
TAMA_208
TAMA_195
TAMO_2
TAMO_91
TAMO_92
TAMO_4
TAMO_19
TAMO_5
TAMO_11
TAMO_9
TAMO_8
TAMO_10
TAMO_16
TAMO_17
TAMO_12
TAMO_14
TAMO_18
TAMO_15
TAMO_13
TAMO_20
TAMO_6
TAMO_7
TAMO_93
TAMO_94
TAMO_95
TAMO_1
TAMO_3
TAMW_179
TAMW_184
C_WALDIN
P_QB_011
C_PAPELI
C_LORENA
C_TRAPP
P_VB_014
P_QP_001
P_QP_003
P_VR_002 and 140 others (0.511)
