# Supplementary material for: Avocado (Persea americana Mill.) Genomic Divergence Across the Victoria, Tanganyika and Rukwa Lake Basins of Tanzania
Source: Int J Mol Sci. 2026 Oct 29;27(21):9699. doi: 10.3390/ijms27219699 (PMC13763250; doi:10.3390/ijms27219699)
Supplement: Supplementary file 1 [file ijms-27-09699-s001.zip › ijms-4614099-supplementary/FigS4_12.pdf]

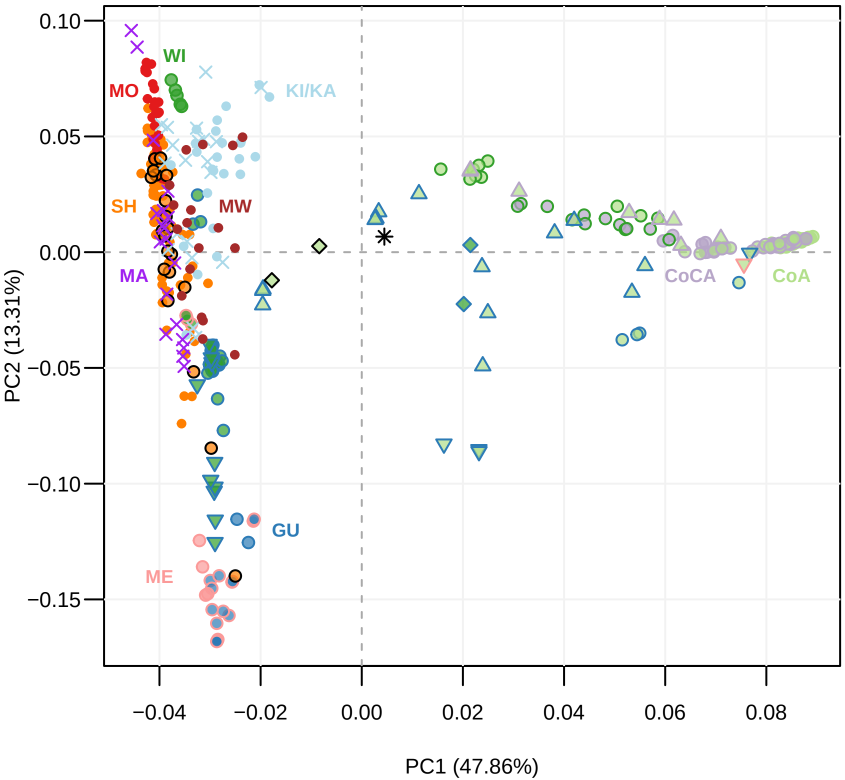

WI
MO
KI/KA
SH
MW
MA
CoCA
CoA
GU
ME
0.10
0.05
0.00
−0.05
−0.10
−0.15
PC2 (13.31%)
−0.04
−0.02
0.00
0.02
0.04
0.06
0.08
PC1 (47.86%)
