# Supplementary material for: Avocado (Persea americana Mill.) Genomic Divergence Across the Victoria, Tanganyika and Rukwa Lake Basins of Tanzania
Source: Int J Mol Sci. 2026 Oct 29;27(21):9699. doi: 10.3390/ijms27219699 (PMC13763250; doi:10.3390/ijms27219699)
Supplement: Supplementary file 1 [file ijms-27-09699-s001.zip › ijms-4614099-supplementary/FigS6_23.pdf]

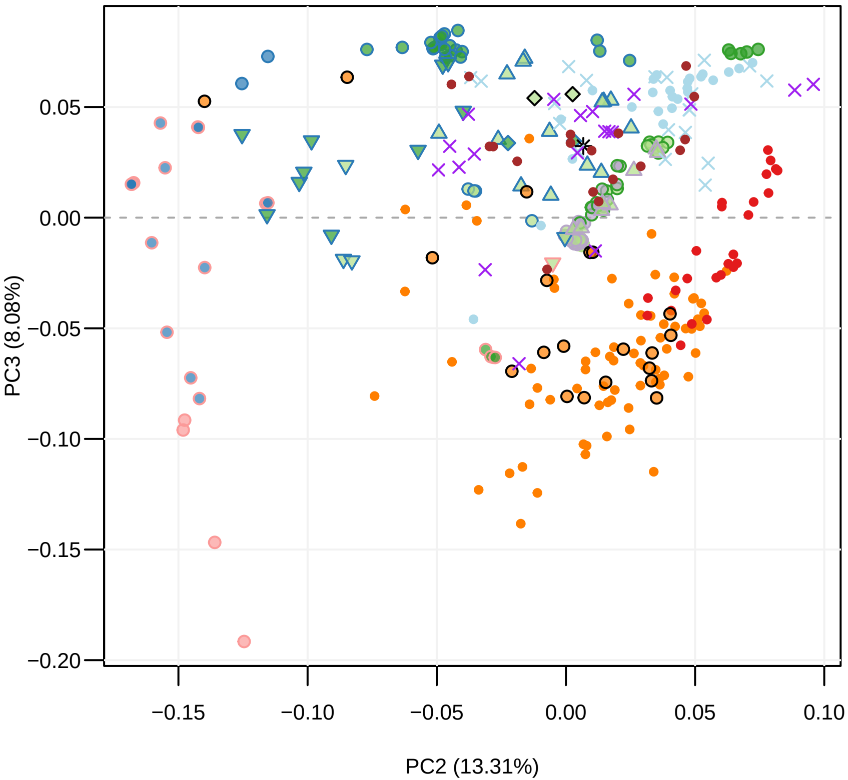

0.05
0.00
−0.05
−0.10
−0.15
−0.20
PC3 (8.08%)
−0.15
−0.10
−0.05
0.00
0.05
0.10
PC2 (13.31%)
