# Supplementary material for: Avocado (Persea americana Mill.) Genomic Divergence Across the Victoria, Tanganyika and Rukwa Lake Basins of Tanzania
Source: Int J Mol Sci. 2026 Oct 29;27(21):9699. doi: 10.3390/ijms27219699 (PMC13763250; doi:10.3390/ijms27219699)
Supplement: Supplementary file 1 [file ijms-27-09699-s001.zip › ijms-4614099-supplementary/FigS8_13.pdf]

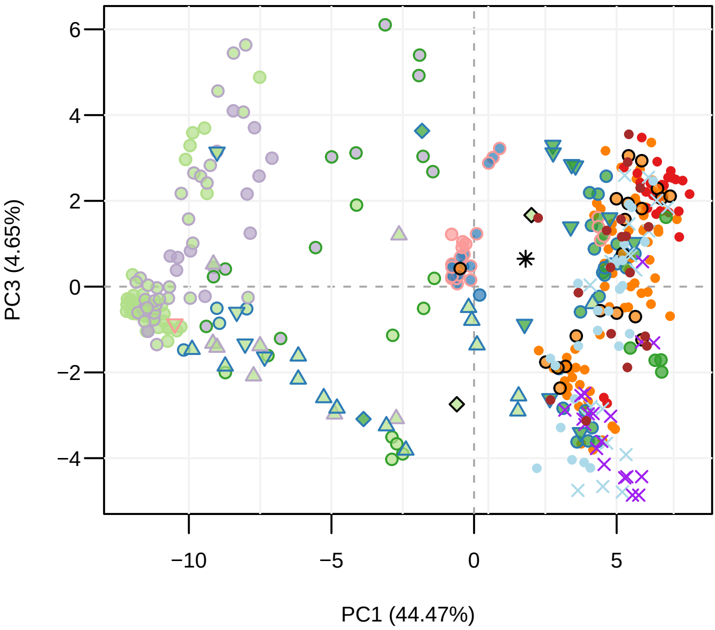

6
4
2
0
−2
−4
PC3 (4.65%)
−10
−5
0
5
PC1 (44.47%)
